# Nidogen-1 expression is associated with overall survival and temozolomide sensitivity in low-grade glioma patients

**DOI:** 10.18632/aging.202789

**Published:** 2021-03-18

**Authors:** Baiwei Zhang, Cheng Xu, Junfeng Liu, Jinsheng Yang, Qinglei Gao, Fei Ye

**Affiliations:** 1 Department of Neurosurgery, Tongji Hospital, Tongji Medical College, Huazhong University of Science and Technology, Wuhan, China; 2 Cancer Biology Research Center, Key Laboratory of the Ministry of Education, Tongji Hospital, Tongji Medical College, Huazhong University of Science and Technology, Wuhan, China; 3 Department of Gynecology and Obstetrics, Tongji Hospital, Tongji Medical College, Huazhong University of Science and Technology, Wuhan, China; 4 Department of Neurosurgery, The First Affiliated Hospital of Henan University of Science and Technology, Luoyang, China

**Keywords:** NID1, glioma, basement membrane, apoptosis, temozolomide

## Abstract

We investigated the prognostic significance of nidogen-1 (NID1) in glioma. Oncomine, GEPIA, UALCAN, CCGA database analyses showed that NID1 transcript levels were significantly upregulated in multiple cancer types, including gliomas. Quantitative RT-PCR analyses confirmed that NID1 expression was significantly upregulated in glioma tissues compared to paired adjacent normal brain tissue samples (n=9). NID1 silencing enhanced *in vitro* apoptosis and the temozolomide sensitivity of U251 and U87-MG glioma cells. Protein-protein interaction network analysis using the STRING and GeneMANIA databases showed that NID1 interacts with several extracellular matrix proteins. TIMER database analysis showed that NID1 expression in low-grade gliomas was associated with tumor infiltration of B cells, CD4^+^ and CD8^+^ T cells, macrophages, neutrophils, and dendritic cells. Kaplan-Meier survival curve analysis showed that low-grade gliomas patients with high NID1 expression were associated with shorter overall survival. However, NID1 expression was not associated with overall survival in glioblastoma multiforme patients. These findings demonstrate that NID1 expression in glioma tissues is associated with overall survival of low-grade glioma patients and temozolomide sensitivity. NID1 is thus a potential prognostic biomarker and therapeutic target in low-grade glioma patients.

## INTRODUCTION

Glioma is a highly malignant brain tumor with poor prognosis despite the availability of conventional therapeutic options including surgical excision followed by adjuvant radio-chemotherapy [1, 2]. Molecular targeted therapy has emerged as an important therapeutic option for several cancers because it provides greater specificity compared to traditional chemo-radiotherapy [3]. However, effective targeted therapies are still not available for glioma patients despite identification of several prognostic biomarkers [4, 5]. Hence, there is an urgent need to identify new and more effective therapeutic targets in gliomas. The abnormally high expression of NID1, which is widely discussed in various types of other cancers including ovarian cancer, non-small cell lung cancer and hepatocellular carcinoma [6–8], in glioma is uncovered through the differently expressed genes (DEGs) analysis between normal and cancer of brain/central nervous system (CNS). However, not many studies on the role of NID1 in glioma are available, and thus we are interested in whether its function in glioma is the same as in other cancer types.

NID1 is a 150 KDa glycoprotein belonging to the Nidogen (also termed entactin) family and is an essential component of the basement membrane [9]. NID1 consists of three (G1-G3) globular domains—one flexible linker domain that connects G1 and G2 domains, and one rod-like domain that separates G2 and G3 domains [10]. NID1 is secreted by mesenchymal cells and deposited between epithelial cells [11, 12]. NID1 is sensitive to various proteases and plays a significant role in basement membrane remodeling [13]. NID2 is a paralog of NID1 with complementary functions [14]. NID1 plays a significant role in the formation and stabilization of the basement membrane network, wound healing, cell adhesion, chemotaxis, and phagotrophy [15–17].

NID1 is also regarded as an oncogene in many cancer types. NID1 promotes epithelial-mesenchymal transition (EMT) and metastasis in ovarian cancer [6]. NID1 enhances adhesion of ETV5-overexpressing endometrial cancer cells to the extracellular matrix (ECM) and promotes their proliferation and migration [18]. Serum NID1 levels are elevated in non-small cell lung cancer (NSCLC) patients compared to the healthy controls [7]. NID1-enriched vesicles secreted by hepatocellular carcinoma (HCC) cells facilitate extrahepatic HCC metastasis [8]. These findings suggest that NID1 plays a crucial role in tumorigenesis. However, the role of NID1 in glioma is not known. Therefore, in this study, we investigated the relationship between NID1 expression and prognosis of glioma patients.

## RESULTS

### NID1 is overexpressed in several cancers

**Figure 1:**
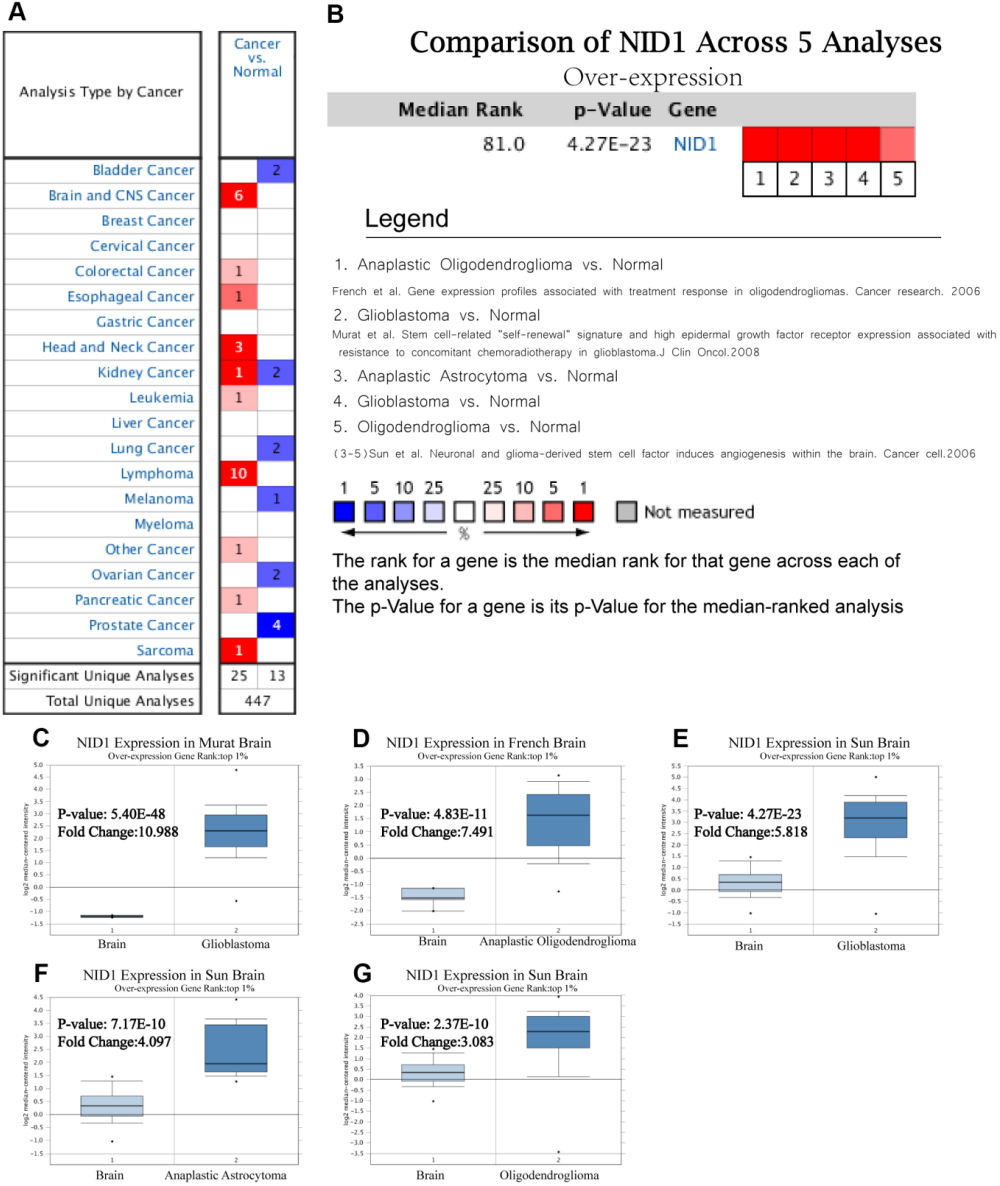
**NID1 is overexpressed in several cancers including different types of glioma.** (**A**) Summary of NID1 expression analyses in multiple cancer types and their corresponding normal tissues. (**B**) Summary of NID1 expression analyses in five studies related to brain and CNS cancers. Note: P< 0.001 indicates statistical significance. Red color indicates high NID1 expression in the corresponding cancer and blue color indicates low NID1 expression in the corresponding cancer. (**C**–**G**) NID1 expression in (**C**) Murat brain (normal brain vs. glioblastoma), (**D**) French brain (normal brain vs. anaplastic oligodendroglioma), (**E**) Sun brain ((normal brain vs. glioblastoma), (**F**) Sun brain (normal brain vs. anaplastic astrocytoma), and (**G**) Sun brain (normal brain vs. oligodendroglioma) datasets are shown. Note: *P*<0.01 indicates statistical significance; NID1 was among the top 1% overexpressed genes in all five different grades of glioma.

We analyzed NID1 expression in 15 different kinds of cancers using the Oncomine database with P ≤ 1E-4, fold change ≥ 2, and gene rank in the top10% as threshold parameters. NID1 expression was significantly higher in multiple cancer types compared to their corresponding normal tissues, as reported in 25 unique cancer studies including 6 studies related to brain and CNS tumors (Figure 1A). The detailed information of the 6 brain tumor-related studies is shown in Table 1. The analysis of 5 studies related to brain and CNS tumors (the sixth study was a TCGA dataset) showed that NID1 was overexpressed in brain tumor tissues compared to the corresponding normal brain tissues (Figure 1B). NID1 was significantly upregulated in glioblastoma (Murat and Sun brain datasets), anaplastic astrocytoma (Sun brain dataset), and oligodendroglioma (French and Sun brain datasets) tissues compared to the corresponding normal tissue samples (Figure 1C–1G).

**Table 1:** NID1 expression data from six studies that analyzed brain and CNS cancer samples.

**Datasets (n)**	**Study object**	**Fold change**	**t**	***P***	***Ref***
Murat Brain (84)	Glioblastoma vs. Normal	10.988	31.646	**5.40E-48**	[22]
French Brain (33)	Anaplastic Oligodendroglioma vs. Normal	7.491	10.191	**4.83E-11**	[23]
Sun Brain (180)	Glioblastoma vs. Normal	5.818	133.786	**4.27E-23**	[24]
	Anaplastic Astrocytoma vs. Normal	4.097	8.582	**7.17E-10**	
	Oligodendroglioma vs. Normal	3.083	7.216	**2.37E-10**	
TCGA Brain (557)	Brain Glioblastoma vs. Normal	9.518	22.006	**7.26E-11**	[-]

### NID1 is overexpressed in several pan-cancer tissue datasets from UALCAN and GEPIA databases

**Figure 2:**
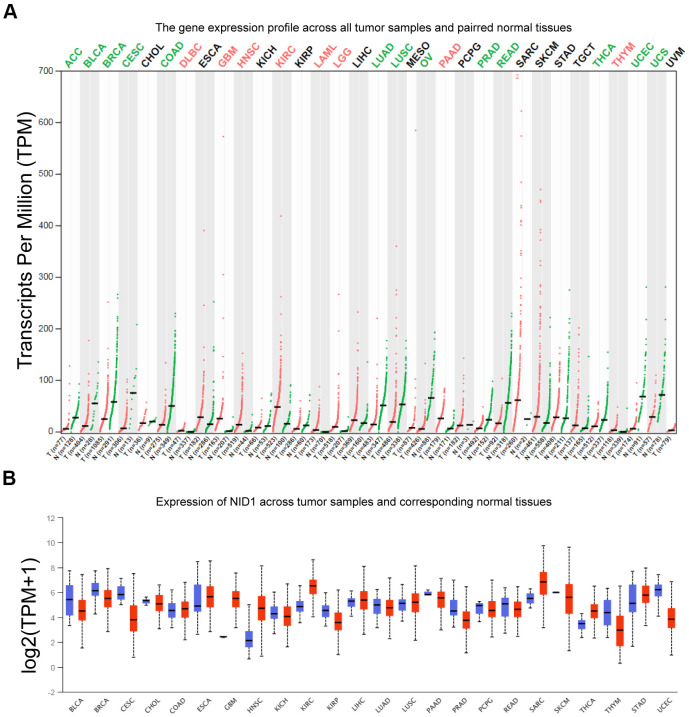
**NID1 expression in pan-cancer tissues from GEPIA and UACLAN databases.** (**A**) NID1 transcript levels in paired tumor and normal tissue samples from the GEPIA database. Red dots represent NID1 expression in tumor samples; green dots indicate NID1 expression in the corresponding normal tissues; black line indicates median NID1 expression; tumor names highlighted in green indicate NID1 downregulation; tumor names highlighted in red indicate NID1 upregulation; tumor names highlighted in black indicate normal NID1 expression; T= tumor tissue; N= normal tissue. (**B**) Validation of NID1 expression levels in different cancers from the UALCAN database. The red boxes represent NID1 expression in tumor tissues and blue boxes represent NID1 expression in the corresponding normal tissues.

We analyzed NID1 expression in pan-cancer tissues using UALCAN and GEPIA databases (Supplementary Table 1). GEPIA database analysis showed that NID1 expression levels were significantly higher in cancers such as low grade glioma, glioblastoma multiforme, kidney renal clear cell carcinoma, head and neck squamous cell carcinoma, and others (Figure 2A). UALCAN database analysis also showed that NID1 expression was significantly higher in several cancer types compared to the corresponding normal tissues (Figure 2B).

### NID1 overexpression in different grades of glioma

**Figure 3:**
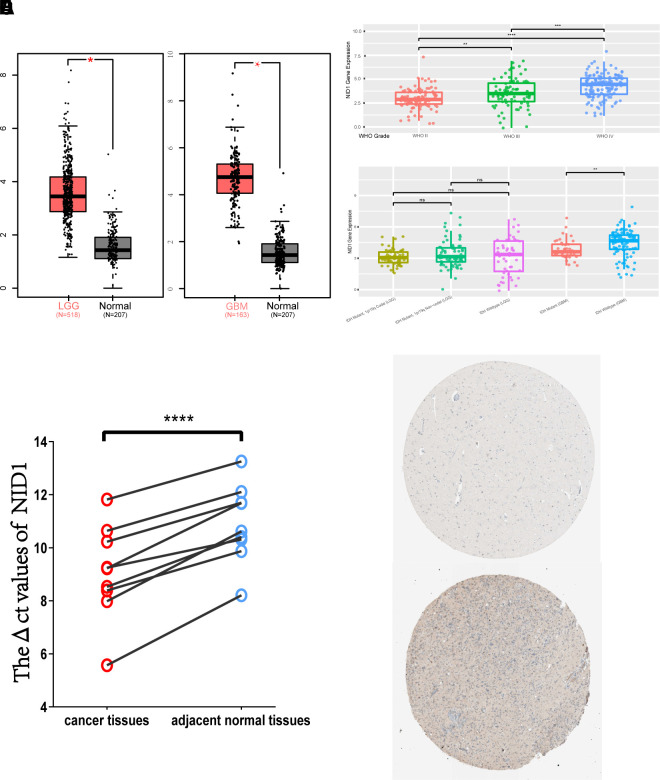
**NID1 mRNA and protein expression in different grades of gliomas.** (**A**) NID1 transcript expression levels in low-grade glioma (LGG; red; n=518), glioblastoma multiforme (GBM; red; n=163) and corresponding normal brain tissues (black; n=207) from the GEPIA datasets. (**B**) NID1 expression levels in different grades of glioma (WHO grades II, III, and IV) from the CCGA dataset. As shown, NID1 expression levels are significantly higher in WHO grades III and IV compared to WHO grade II. (**C**) NID1 expression levels in LGG and GBM patients belonging to IDH mutant and wild-type genotypes from the CCGA datasets. As shown, IDH mutant GBM patients show lower NID1 levels compared to the IDH wild-type GBM patients. NID1 levels in IDH mutant and wild-type LGG patients are comparable and not statistically significant. (**D**) NID1 expression is upregulated in all 9 glioma tissue samples compared to their corresponding normal brain tissue samples. Red circles represent glioma tissues and blue circles represent normal brain tissues. Higher ΔCt value represents lower NID1 expression. (**E**) Representative IHC-stained brain section images from the HPA database show NID1 expression in normal healthy individual (patient i.d. 2521) and glioma patient (patient id: 3092). Blue staining represents anti-NID1 antibody staining. Note: * P<0.05;** P<0.01;*** P<0.001;**** P<0.0001.

Next, we analyzed the expression of NID1 in various grades of gliomas. According to the World Health Organization (WHO) criteria, gliomas are classified into four grades—WHO grades I, II, III, and IV [19]. WHO grades I, II, and III are collectively called as low-grade gliomas (LGG), whereas, WHO grade IV is called glioblastoma multiforme (GBM) and is the most malignant type of glioma [20, 21]. GEPIA database analysis showed that NID1 transcript expression in the LGG and GBM tissues was significantly higher compared to the normal brain tissues (Figure 3A). CGGA database analysis also showed that NID1 expression was significantly increased in the higher glioma grades (Figure 3B). Furthermore, IDH mutant GBM tissues showed significantly lower NID1 expression than the IDH wild-type GBM tissues, but NID1 expression in IDH mutant and wild-type LGG tissues was similar (Figure 3C). Furthermore, qRT-PCR analysis of 9 pairs of glioma tissues and adjacent non-tumor brain tissues (Supplementary Table 2) showed that NID1 mRNA expression was significantly higher in 100% (9/9) of glioma tissues compared to the corresponding adjacent normal brain tissues (p<0.0001; Figure 3D).

### NID1 protein expression in brain tissues of glioma patients

Next, we analyzed NID1 protein expression levels in the brains of normal healthy individuals and glioma patients using the HPA database. NID1 protein expression in the normal healthy adult brain tissue (for example, patient id: 2521) was moderate and restricted to the ECM-neuropil area and not detected in the endothelial cells, glial cells, and neuronal cells (Figure 3E; top). In the glioma patients (patient id: 3092) however, heterogeneous cytoplasmic or membranous NID1 protein expression was observed in the ECM area of both the normal brain neuropils and the tumor cells (Figure 3E-bottom). Moreover, NID1 protein staining was positive in the highly malignant glioma cells, but the staining intensity in the glioma cells (75%-25%) was lower compared to the normal neuropil area (>75%; Figure 3E-bottom). These results suggested that NID1 may be involved in the ECM remodeling around the glioma tissue.

### Silencing of NID1 promotes apoptosis and TMZ sensitivity of U87-MG and U251 glioma cells

**Figure 4:**
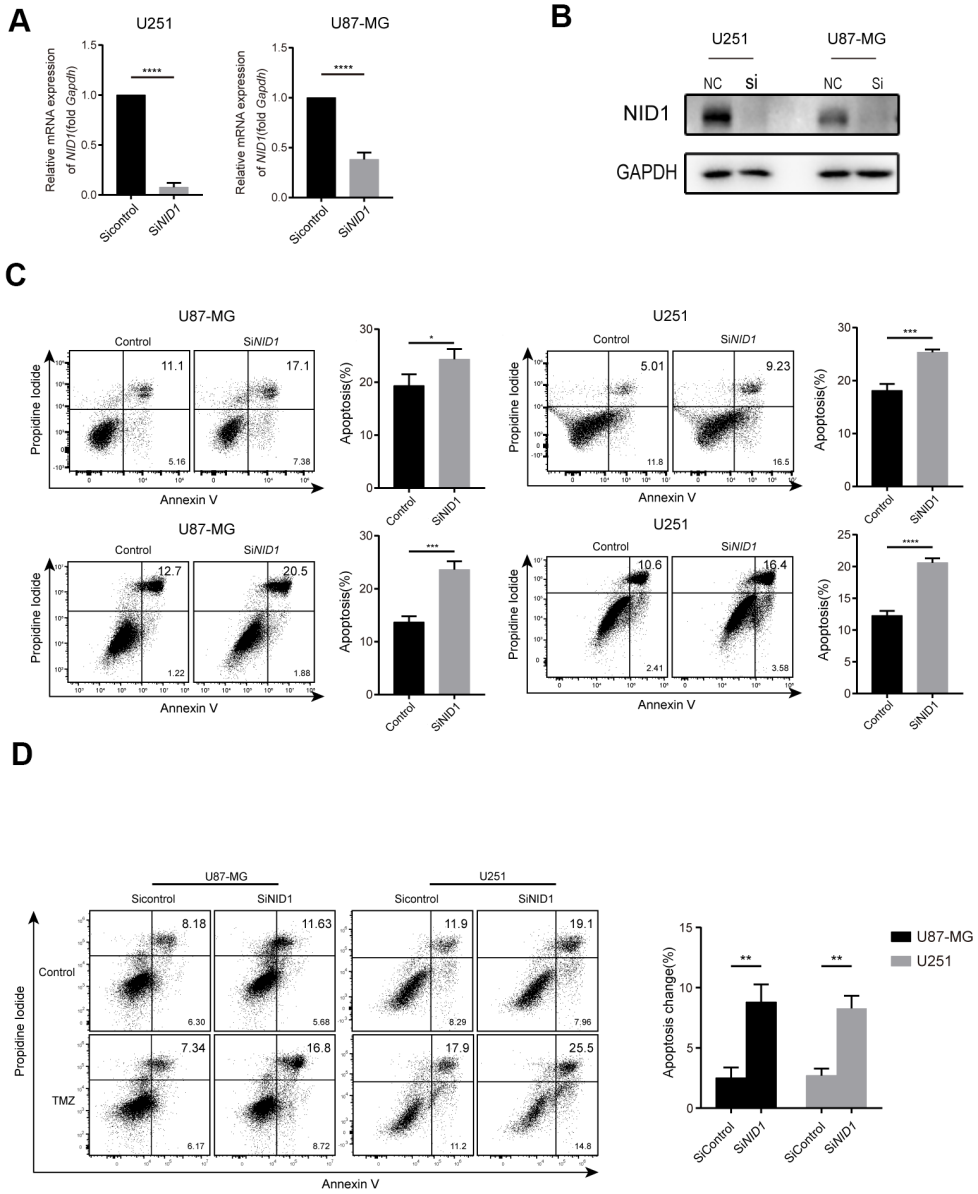
**NID1 silencing in U87-MG and U251 glioma cells enhanced apoptosis and sensitivity to TMZ.** (**A**) QRT-PCR analysis shows NID1 mRNA levels in si-NID1- and si-NC-transfected U251 and U87-MG glioma cell lines. (**B**) Representative western blot shows NID1 protein levels in si-NID1- and si-NC-transfected U251 and U87-MG glioma cell lines. (**C**) Representative FACS plots and histograms show percentage apoptosis in si-NID1- and si-NC-transfected U251 and U87-MG glioma cells based on AnnexinV-FITC/PI staining. (**D**) Representative FACS plots and histograms show percentage apoptosis in si-NID1- and si-NC-transfected U251 and U87-MG glioma cells treated with or without TMZ. As shown, NID1 silencing improved temozolomide (TMZ) sensitivity in glioma cells. Note: * *P*<0.05;** *P*<0.01;*** P<0.001;**** P<0.0001.

NID1 is a glycoprotein that is required for the stability of the basement membrane [25]. Therefore, we analyzed if NID1 regulates glioma cell apoptosis. RT-PCR and western blot analysis confirmed that NID1 expression was significantly reduced in the si-NID1-transfected U87-MG and U251 glioma cells compared to the corresponding si-NC-transfected glioma cells (Figure 4A, 4B). The apoptotic rate was significantly increased in the NID1-silenced U87-MG and U251 glioma cells compared to the corresponding si-NC-transfected controls (P<0.05, Figure 4C). Moreover, we also compared the apoptosis rate between si-NID1 group and si-NC group with or without TMZ treatment, a significant increase of apoptosis rate in si-NID1 group was revealed compared to si-NC group (P<0.05, Figure 4D), indicating that NID1-silencing could be an effective approach to increased TMZ sensitivity of glioma cells. Hence, this suggested that NID1 is a potential therapeutic target in glioma.

### Functional enrichment analysis of top 100 NID1-related genes

**Figure 5:**
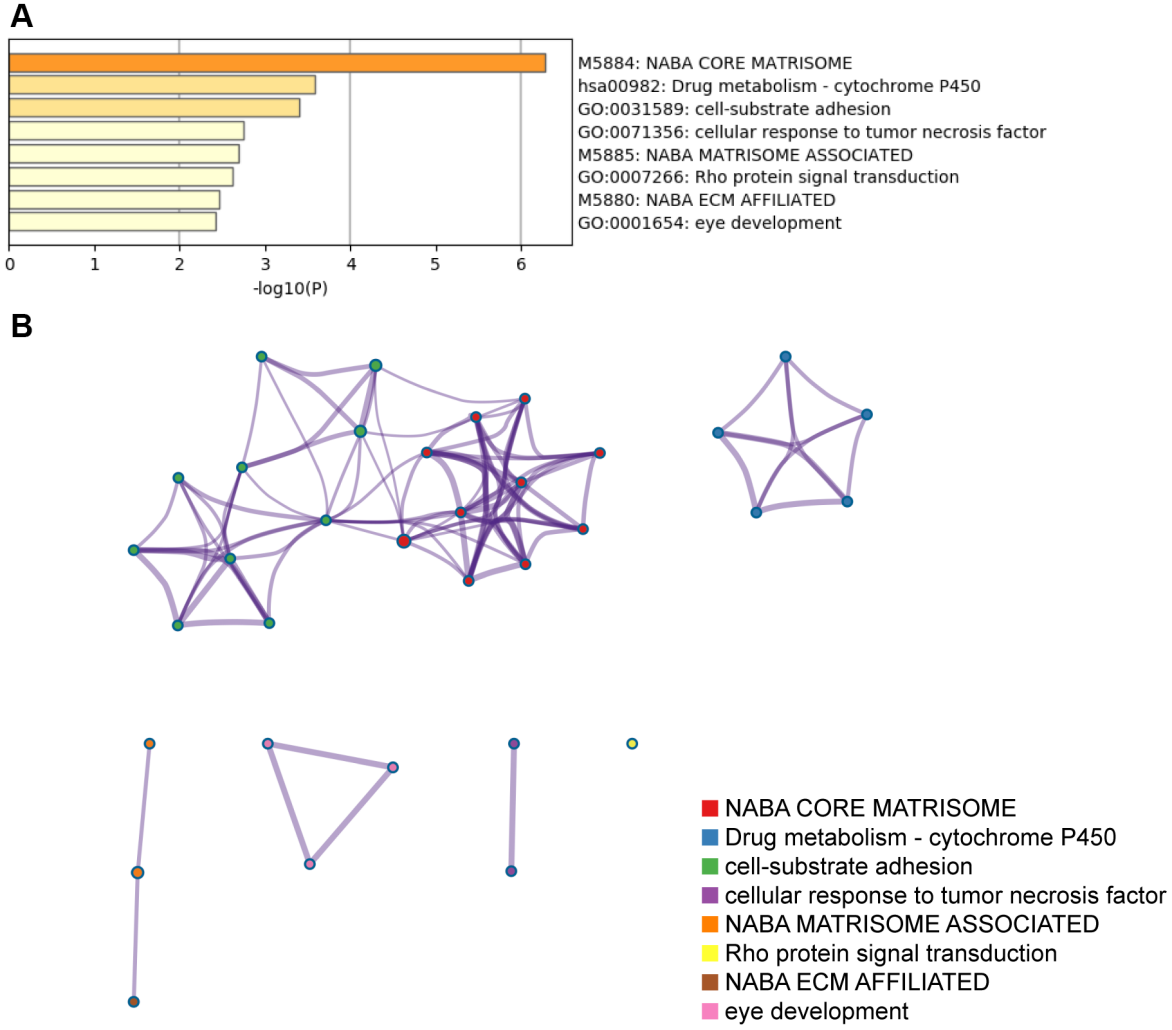
**The top GO terms and pathways related to top 100 NID1-related genes in GBM tissues.** (**A**) Heatmap shows top GO terms and (KEGG/Canonical) pathways related to the top 100 NID1-related genes expressed in GBM tissues. The -log_10_P values are plotted on the X-axis. (**B**) The network of enriched gene sets representing the top 100 NID1-related genes expressed in GBM tissues.

We performed gene ontology (GO) and pathways (KEGG/ Canonical) analyses of the top100 NID1-related genes identified from the GBM data in the GEPIA database (Supplementary Table 3). NID1-related genes were enriched in GO terms (biological processes) and pathways related to cell-substrate adhesion, cellular response to tumor necrosis factor, Rho protein signal transduction, eye development, drug metabolism– Cytochrome P450 (KEGG pathway), NABA core matrisome, NABA matrisome associated, and NABA ECM affiliated gene sets (Canonical pathway; Figure 5A, 5B).

### PPI network analysis of NID1 and its interacting partners

**Figure 6:**
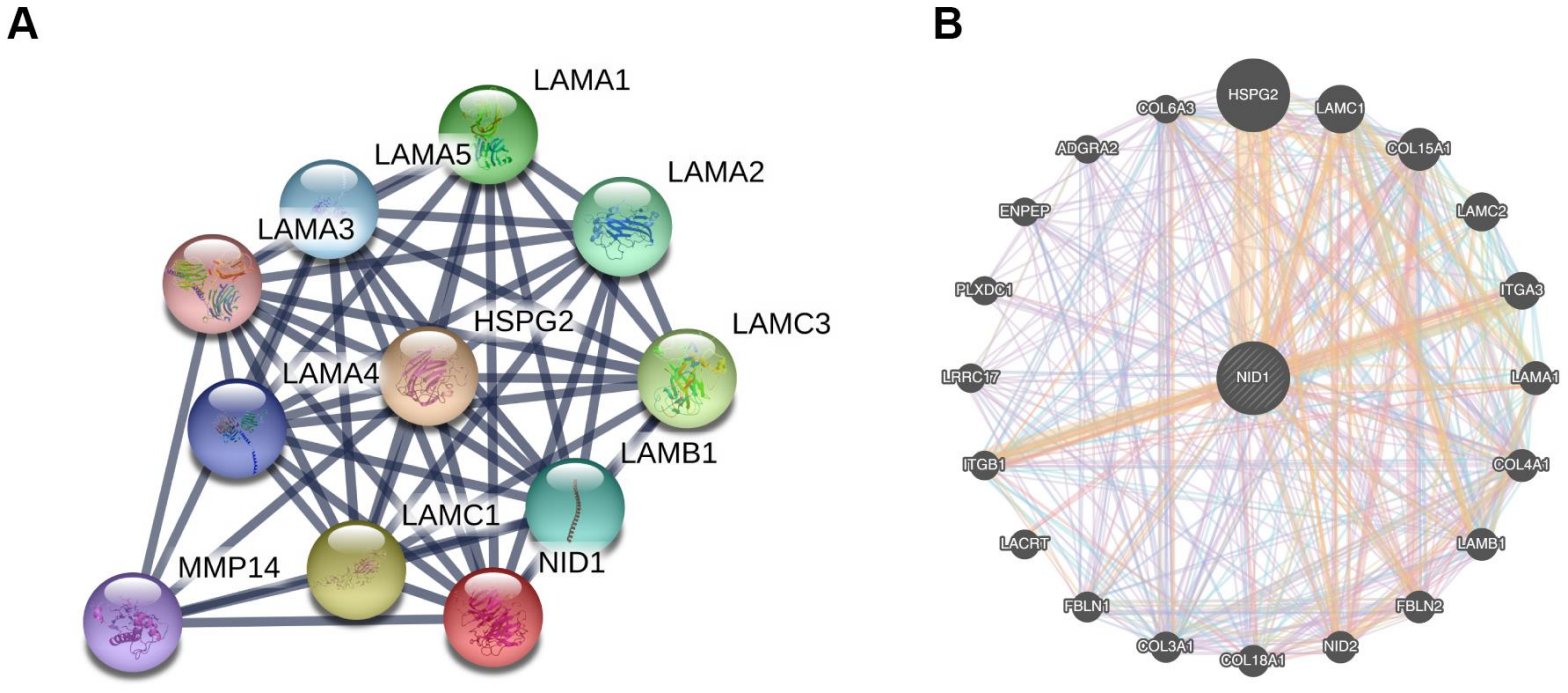
**PPI networks of NID1 and its interacting protein partners.** (**A**) PPI network constructed using the STRING database shows NID1 and the NID1-interacting proteins. The line thickness indicates strength of interaction between any two proteins. (**B**) GeneMANIA database analysis shows that NID1 interacts with ECM proteins such as HSPG2, LAMC1, and others.

We used the STRING database to construct a protein-protein interaction (PPI) network of NID1-associated proteins that included proteins such as HSPG2, matrix metalloproteinase (MMP), and laminin subunit proteins such as LAMA1, LAMC1, LAMB1, and others (Figure 6A). Furthermore, GeneMANIA database analysis showed that NID1 interacted with NID2 (paralog of NID1), collagen subunits such as COL15A1, COL4A1, COL18A1, and COL3A1, and integrins such as ITGA3 and ITGB1, all of which were components of the basement membrane (BM) or extracellular matrix (ECM) (Figure 6B).

### NID1 expression is associated with immune cell infiltration in glioma

**Figure 7:**
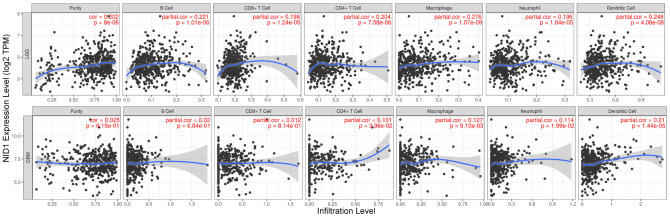
**Higher NID1 expression correlates with increased tumor infiltration of multiple immune cell types.** TIMER database analysis shows correlation between NID1 expression levels in LGG (top) and GBM (bottom) patient tissues and tumor infiltration levels of immune cell types, namely, B cells, CD8+ T cells, CD4+T cells, macrophages, neutrophils, and dendritic cells. Each dot corresponds to a glioma patient (LGG or GBM). The blue line represents median levels of tumor-infiltrated immune cells.

Basement membrane is a thin layer of the extracellular matrix (ECM) that is required for cellular function and tissue integrity [26]. In cancers, the extracellular tumor microenvironment, participates in the development and progression of tumors [27, 28]. Therefore, we analyzed the relationship between NID1 expression levels and the status of immune cell infiltration in LGG and GBM tissues using the TIMER database. The results showed significant correlation between NID1 expression levels and the infiltration levels of immune cells such as B cells, CD8^+^ T cells, CD4^+^ T cells, macrophages, neutrophils, and dendritic in LGG tissues, however, which is unobvious in GBM tissues. (Figure 7).

### High NID1 expression correlates with poor overall survival of LGG patients

**Figure 8:**
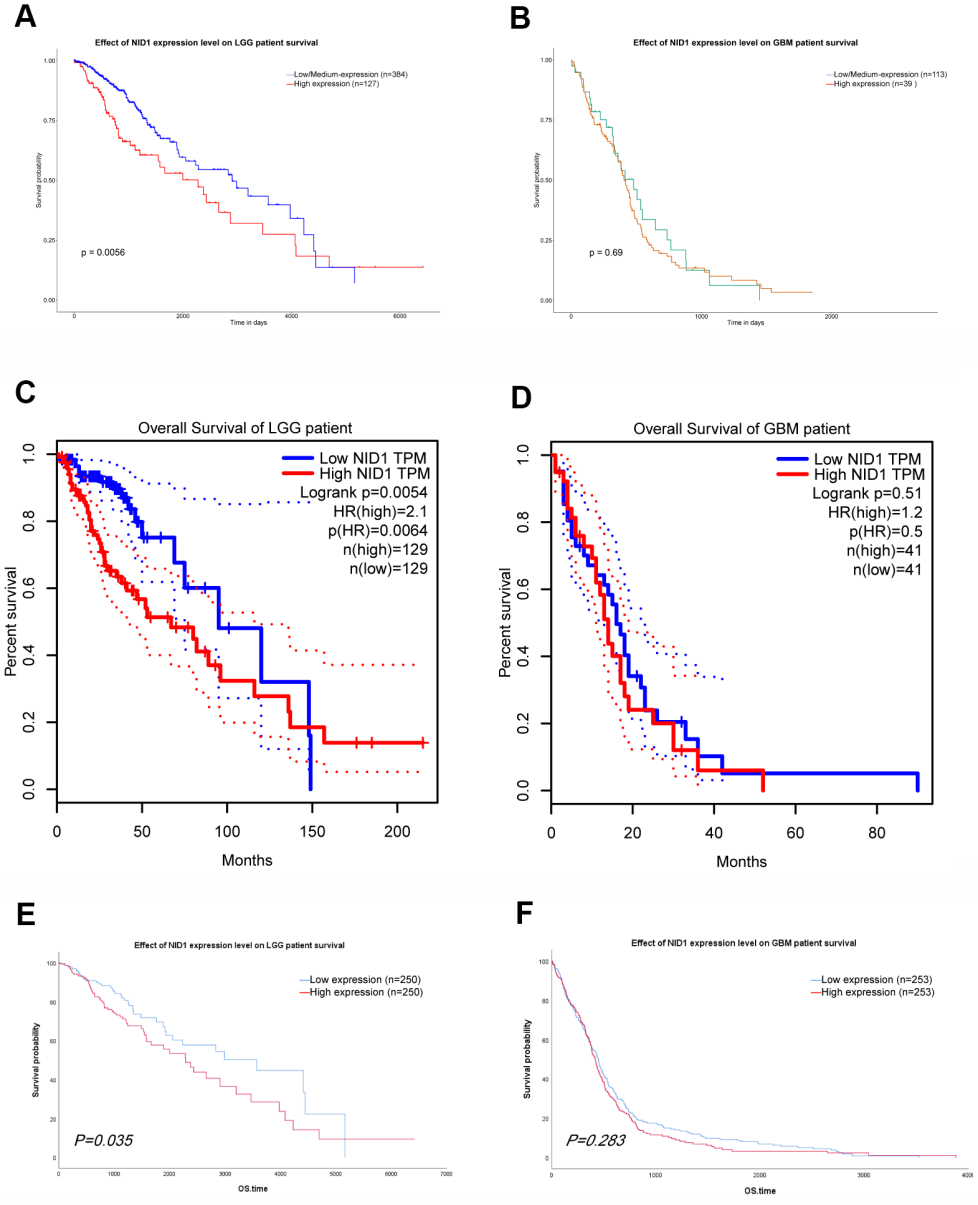
**NID1 expression correlates with overall survival of LGG patients.** (**A**, **B**) Kaplan-Meier survival curves show overall survival of low- and high-NID1-expressing LGG and GBM patients from the UALCAN database. (**C**, **D**) Kaplan-Meier survival curves show overall survival of low- and high-NID1-expressing LGG and GBM patients from the GEPIA database. HR refers to hazard ratio. (**E**, **F**) Kaplan-Meier survival curves show overall survival of low- and high-NID1-expressing LGG and GBM patients from the TCGA database. Note: Blue represents low NID1 expression; red represents high NID1 expression.

Next, we evaluated the prognostic value of NID1 expression in glioma patients from the UALCAN and GEPIA databases. Kaplan-Meier survival curve analysis showed that high NID1 expression correlated with worse overall survival of LGG patients (Figure 8A, 8C). However, we did not observe significant correlation between NID1 expression levels and overall survival of GBM patients (Figure 8B, 8D). Furthermore, we analyzed the survival data of glioma patients in the TCGA database (UCSC Xena browser) after excluding cases of tumor recurrence tumor and those without survival time. We sub-divided TCGA-glioma patients into high- and low-NID1 expression groups based on median NID1 expression levels. Kaplan–Meier survival curve analysis showed that overall survival (OS) of LGG patients with low NID1 expression was significantly higher compared to LGG patients with high NID1 expression (P = 0.035; Figure 8E). However, NID1 expression levels did not correlate with survival in GBM patients (P=0.283; Figure 8F). These results suggested that high NID1 expression was associated with worse OS in LGG patients.

## DISCUSSION

Basement membranes are thin sheets of specialized extracellular matrix (ECM) that provide structural support to cells and tissues, and also regulate cellular proliferation, adhesion, migration, differentiation, and survival [25, 26, 29–31]. Basement membranes play a significant role in cellular signaling, normal growth and development of tissues and organs, and human diseases including cancers [32]. The basement membrane consists of various glycoproteins and proteoglycan protomers including laminin, collagen IV, heparan sulphate proteoglycans such as perlecan and agrin, nidogen/entactin, fibulin like BM-90, and BM-40 (basement membrane protein of 40 kDa) [25, 26, 29, 33]. Laminins are heterotrimeric proteins that form a cross shaped structure with one long arm and three short arms, and are made up of one of the five α chains, one of the three β chains, and one of the three γ chains; the N-terminal regions of the α, β, and γ chains (the short arm) assemble to form ternary nodes that are involved in forming the cell-associated network [34–37]. Collagen IV (type IV collagen) is only found in the basement membranes and consists of six highly homologous but genetically distinct α-chains (α1 to α6), an N-terminal 7S domain, a central collagenous triple helix domain, and a C-terminal non-collagenous (NC1) domain [38]. The α-chains intertwine into a triple helix through the collagenous domain that contains Gly-X-Y amino-acid triple repeats; collagen IV molecules assemble into networks through their N-terminal and C-terminal end-domains [39–41]. The laminin- and collagen IV- networks in the basement membranes are connected with each other and stabilized by NID1 and other basement membrane proteins [25, 32, 42, 43]. Heparin sulfate proteoglycans (HSPGs) are glycoproteins with one or more heparin sulfate chains; basement membranes contain perlecan and agrin HSPGs, which increase the volume of extracellular matrix [32]. Tysnes et al. reported that *in vitro* glioma cell migration was stimulated by laminin, fibronectin, and collagen type IV [44]. Ohlund et al. reported that high circulating levels of collagen type IV in post-operative pancreatic cancer patients were associated with rapid relapse and poor survival rates [45]. Agrin plays a significant role in maintaining the blood-brain barrier (BBB), and its deficiency leads to brain edema in GBM patients [46].

NID1 is expressed in normal brain tissues and is required for maintaining normal synaptic plasticity and network excitability [47]. Nonsense mutations in the NID1 gene are associated with autosomal dominant Dandy-Walker malformation and occipital cephaloceles (ADDWOC), a disorder characterized by normal neurological development but variable cerebellar hypoplasia, meningeal anomalies, and occipital skull defects [48–50]. NID1 also promotes EMT and metastasis in ovarian cancer [6]. Moreover, cathepsin degradation of Nid-1 is strongly associated with NSCLC [7]. Mao et al. reported that NID1-enriched extracellular vesicles derived from HCC cells facilitate colonization of tumor cells and extra-hepatic metastasis by activating pulmonary fibroblasts to secrete TNFR1 [8]. Our study showed that NID1 expression was significantly higher in glioblastoma, anaplastic astrocytoma, and oligodendroglioma compared to the normal brain tissues and cells using glioma datasets from Oncomine, UALCAN, and GEPIA databases. This data was confirmed by qRT-PCR analysis of 9 paired glioma and normal brain samples. We also showed that NID1 silencing induced glioma cell apoptosis and increased sensitivity of glioma cells to TMZ. These data suggest that NID1 plays a key role in glioma cell survival and TMZ resistance, although the underlying mechanisms are not known and require further investigations.

Immunohistochemical staining data in the HPA database showed that NID1 staining in the brain tissues of healthy individuals and patients with high-grade glioma was restricted to the ECM area. Furthermore, NID1 protein levels in highly malignant glioma were significantly lower than in the normal neuropils. That is because the interactions between ECM and the glioma cells are highly complex [51]. Glioblastoma multiforme (GBM; WHO grade IV astrocytoma) is the most malignant astrocyte tumor [52]. A widely accepted speculation is that malignant tumors possess a less differentiated or a progenitor state of their cellular origin [53], which may result in loss of primary brain cells components and structural features, like BM dissociation. Furthermore, MMPs protein levels in brain glioma tissue are elevated with the increasing pathological grades [54–56]. MMPs promote glioma cell infiltration by cleaving glycoproteins and adhesion proteins in the ECM [57–59]. Nidogen is hypersensitive to proteolytic cleavage and is protected from hydrolysis by its interaction with laminin [13, 15, 60]. The weak NID1 protein staining in the brain tissue of glioma patients suggests that NID1 is cleaved during glioma progression. Hence, our data suggests that NID1 regulates malignant transformation of glioma because it maintains basement membrane integrity.

We constructed PPI network to identify NID1-interacting proteins. NID1 interacted with several ECM components such as laminin, HSPG2 (Perlecan), and MMPs. The amino acid sequences of murine nidogen suggest that it binds to laminin, collagen IV, and cells through multiple domains [61]. Aumailley et al. demonstrated that nidogen is an integral part of the ternary complex between laminin and collagen IV in the basement membrane [62]. High-affinity binding of NID1 to laminin γ1 chain is critical for the formation of basement membrane [63]. Halfter et al. reported that deletion of NID1 binding site in laminin γ1 chain caused defects in the pial basement membrane and disrupted neuronal migration and proper cortical development [64]. NID1 also played an indirect role in integrin signaling by being an integral part of the laminin-NID1 complex that anchored α7β1 integrin, one of the cell surface integrin receptors [65]. The high-affinity interaction between NID1 and Perlecan (HSPG2) also contributed to basement membrane function and stabilization [66, 67]. Titz et al. demonstrated that MMP-19 preferentially cleaved out the G3 globular domain of NID1, which contained the binding site for the γ1 chain of laminin-1 and collagen IV, thereby abolishing its ability to cross-link ECM proteins [68]. Hence, NID1 plays an integral role in extracellular signaling and structural integrity of ECM.

We also demonstrated that LGG patients with low NID1 expression showed significantly higher OS rates. However, NID1 expression did not correlate with OS of GBM patients. Since interactions between ECM components and cells are complex, further studies are necessary to investigate the distinct roles of NID1 in LGG and GBM.

In conclusion, our study shows that NID1 is up-regulated in glioma tissues. Functional enrichment and PPI network analyses demonstrate that NID1 interacts with several ECM proteins, thereby confirming its role in stabilizing the basement membrane. NID1 silencing promotes *in vitro* apoptosis and TMZ sensitivity of glioma cells. Furthermore, low NID1 expression in LGG patients correlates with significantly higher overall survival. These findings suggest that NID1 plays a significant role in glioma development and is a potential therapeutic target in glioma patients.

## MATERIALS AND METHODS

### Bioinformatics analysis

The Oncomine (https://www.oncomine.org) database [69] was used to analyze NID1 transcript levels in several cancer types including gliomas and their corresponding normal tissues with P-value <0.05 and fold change ≥2 as threshold parameters.

Gene Expression Profiling Interactive Analysis (GEPIA; http://gepia.cancer-pku.cn/) is a comprehensive web-based tool that provides interactive and customizable functions to analyze differential gene expression, patient survival, similar gene filtering, and correlation analysis using RNA sequencing data from TCGA and GTEx datasets [70]. We used the GEPIA database to evaluate NID1 expression profiles in several tumors, identify top 100 genes that correlate with NID1 expression, and compare survival of glioma patients with high and low NID1 expression.

UALCAN (http://ualcan.path.uab.edu) is a comprehensive and interactive web portal with TCGA level 3 RNA-seq and clinical data for 31 cancer types, and is used to determine differential expression and prognostic value of target genes in cancer [71]. In this study, we used the UALCAN database to verify NID1 expression in pan-cancer tissues and evaluate prognostic value of NID1 in glioma patients.

Chinese Glioma Genome Atlas (CGGA) (http://www.cgga.org.cn/) is a user-friendly database with functional genomic data for approximately 2,000 primary and recurrent glioma samples from Chinese cohorts. The analysis tools in the CGGA database allow access to gene mutational data, mRNA and microRNA expression data, DNA methylation profiles, as well as survival and correlation analysis of specific glioma types [72]. We used the CGG database to analyze NID1 expression levels in different glioma grades.

Human Protein Atlas (HPA) (https://www.proteinatlas.org) is a database that contains maps of all known human proteins in cells, tissues, and organs by integration of data from various omics technologies, including antibody-based imaging, transcriptomics, systems biology, and mass-spectrometry-based proteomics [73–76]. In this study, we used HPA database to determine NID1 protein expression in gliomas and normal brain tissues.

Metascape (http://metascape.org) is a web-based portal that combines functional enrichment, interactome analysis, gene annotation, and membership search from over 40 independent knowledge-bases into a single integrated portal [77]. We performed Gene Ontology (GO) and Kyoto Encyclopedia of Genes and Genomes (KEGG) pathway analyses using this portal to comprehensively annotate a list of 100 NID1-related genes.

We constructed Protein-Protein interaction (PPI) networks of NID1 and its interacting proteins using STRING and GeneMANIA databases. The STRING database (http://string.embl.de/) comprehensively integrates protein-protein interaction information from all the available sources complemented with computational predictions in order to achieve a comprehensive global network that includes direct and indirect protein interactions [78, 79]. Gene MANIA (http://www.genemania.org) is a user-friendly web platform to find functionally similar genes using extensive genomic and proteomic data [80].

Tumor Immune Estimation Resource (TIMER) (http://cistrome.shinyapps.io/timer/) database is used to characterize association between tumor infiltration of several immune cell types and other disease-related factors including gene expression, clinical outcomes, somatic mutations, and somatic copy number alterations [81]. In this study, we analyzed the association between NID1 expression levels and infiltration status of six different immune cell types in gliomas (LGG and GBM) after adjusting for tumor purity using Spearman correlation coefficient (COR) and P-values. COR>0 indicated positive correlation and COR<0 indicated negative correlation; P <0.05 was considered statistically significant.

We also downloaded RNA sequencing data and corresponding clinical information of the glioma patient samples from The Cancer Genome Atlas (TCGA) database using the University of California Santa Cruz (UCSC) Xena browser (http://xena.ucsc.edu/) and analyzed prognostic significance of NID1 gene expression with OS of glioma patients by evaluating Kaplan-Meier survival curves [82].

### Glioma cell lines and cell culture

The U87-MG and U251 glioma cell lines were purchased from American Type Culture Collection (ATCC, Rockville, MD, USA) and cultured in Dulbecco’s Modified Eagle Medium (DMEM; Gibco, NY, USA) containing 10% fetal bovine serum (FBS; BI, Israel), penicillin (100 units/ml), and streptomycin (100 μg/ml) in a humidified incubator maintained at 5% CO_2_ and 37° C.

### NID1 silencing and temozolomide sensitivity

We purchased NID1-specific and non-specific control (NC) siRNAs from TsingKe Biotechnology (Beijing, China) and transfected them into glioma cell lines using lipofectamine 2000 (Invitrogen, USA) according to the manufacturer’s instructions. We also tested temozolomide (TargetMol; Target Molecule Corp, Boston, MA, USA) sensitivity of siNID-1- and si-NC-transfected glioma cells. In brief, groups of si-NID1- and si-NC-transfected glioma cells were treated with TMZ (200μM, 24 hours), after that, the apoptosis rates of si-NID1 group and si-NC group with or without TMZ treatment were calculated respectively.

### Glioma patient samples

We obtained fresh glioma and adjacent normal brain tissue samples from 9 patients that underwent surgery at the Department of Neurosurgery, Tongji Hospital, Wuhan. We obtained signed written consent from all patients. This study was conducted according to the protocol and guidelines approved by the Tongji hospital Institutional Review Board. The samples were immediately incubated with Trizol (Takara Bio, Japan) at 0° C in a specimen-box and stored at -80° C. Total RNA was extracted within 12 h after sample isolation.

### Western blot

The si-NID1 and si-NC transfected glioma cells were lysed in pre-chilled radioimmunoprecipitation (RIPA) buffer (Servicebio, Wuhan, China) containing protease inhibitor PMSF (1:100) and phosphatase inhibitor cocktail (1:100) at 0° C for 30 min. The protein concentrations were measured using BCA protein assay kit (Beyotime, Shanghai, China). Equal amounts of protein samples (40 μg) were separated by 10% SDS-PAGE and transferred onto PVDF membranes. The membranes were blocked with 5% bovine serum albumin (BSA) for 1 h at room temperature and incubated overnight at 4° C with primary antibodies against NID1 (1:1000;AF2570-SP; R&D Systems, MN, USA) and GAPDH (1:1000;ab9485;Abcam, UK). Then, the membranes were washed thrice with TBST buffer (25 mM Tri-HCl, pH7.5, 137 mM NaCl, 2.7 mM KCl, and 0.05% Tween-20) and incubated with HRP-conjugated anti-goat IgG (1:3000; Servicebio, Wuhan, China) or HRP-conjugated anti-rabbit IgG (1:5000; Antgene, Wuhan, China) for 1 h at room temperature. The membranes were then washed thrice with TBST. The blots were developed using the Enhanced chemiluminescence kit (ECL, Bio-Rad, CA, USA). The protein bands were quantified by Image J software.

### Real-time quantitative polymerase chain reaction

Total RNA of primary glioma and adjacent normal brain tissue samples were extracted with Trizol (Takara Bio, Japan) according to the manufacturer’s protocols. Then, cDNAs were synthesized from 10 μl of total RNA using HiScript^®^ II Q RT SuperMix Kit (Vazyme, Nanjing, China). Quantitative real-time PCR was performed in the CFX Connect Real-Time System (Bio-Rad Laboratories, Berkeley, CA, USA) using ChamQ^TM^ Universal SYBR^®^ qPCR Master Mix (Vazyme, Nanjing, China) according to the manufacturer’s instructions. The qPCR primer sequences were as follows:

Human NID1 (forward), 5’-GACAGCGTGTTCGTCCTGTA-3’; Human NID1 (reverse), 5’-ACACCTCCCGATGGTCAAAAG-3’; Human GAPDH (forward), 5’-GCATCCTGGGCTACACTGAG-3’; Human GAPDH (reverse), 5’-TAACGGGAGTTGCTGGTGAA-3’.

The samples were analyzed independently thrice. The real-time PCR protocol was: 1 cycle of 95° C for 90 s followed by 40 cycles of 95° C for 10 s and 60° C for 60 s. The mean cycle threshold (Ct) values for NID1 (target gene) and GAPDH (endogenous control) were determined. Relative levels of NID1 mRNA expression were quantified using the 2−^ΔΔCt^ method.

### Apoptosis assay

The glioma cells after treatments were washed twice in pre-chilled PBS, resuspended in 100 μl of binding buffer, and stained with the Annexin V-FITC/propidium iodide kit (BD Biosciences, NJ, USA) in the dark according to manufacturer’s recommendations. Then, the samples were analyzed in a FACS Calibur (Beckman Coulter, USA). We collected a minimum of 10,000 cells for each sample. The percentages of apoptotic cells (Annexin V-FITC^+^ PI^+^ plus Annexin V-FITC^+^ PI^-^ cells) were determined using the FlowJo software (Treestar, Ashland, OR).

### Statistical analysis

The data was analyzed using paired student’s t-test and chi-square test. Statistical analysis was performed using GraphPad Prism 8.0 (GraphPad Software, CA, USA) and SPSS statistical software version 25.0 (IBM, Armonk, NY, USA). Kaplan-Meier survival curve analysis was used to evaluate overall survival of glioma patients with high and low NID1 expression. The differences between groups were assessed by log-rank test. P<0.05 was considered statistically significant. All experiments were repeated at least thrice.

## Supplementary Material

Supplementary Tables
