# Supplementary material for: Nidogen-1 expression is associated with overall survival and temozolomide sensitivity in low-grade glioma patients
Source: Aging (Albany NY). 2021 Mar 18;13(6):9085–107. doi: 10.18632/aging.202789 (PMC8034893; doi:10.18632/aging.202789)
Supplement: Supplementary Tables [file aging-13-202789-s001.pdf]

SUPPLEMENTARY TABLES

Supplementary Table 1. List of cancer types included in this study.

ACC	Adrenocortical carcinoma;
BLCA	Bladder urothelial carcinoma;
BRCA	Breast invasive carcinoma;
CESC	Cervical squamous cell carcinoma;
CHOL	Cholangiocarcinoma;
COAD	Colon adenocarcinoma;
DLBC	Lymphoid Neoplasm Diffuse Large B-cell Lymphoma;
ESCA	Esophageal carcinoma;
GBM	Glioblastoma multiforme;
HNSC	Head and Neck squamous cell carcinoma;
KICH	Kidney Chromophobe;
KIRC	Kidney renal clear cell carcinoma;
KIRP	Kidney renal papillary cell carcinoma;
LGG	Brain lower grade glioma;
OV	Ovarian serous cystadenocarcinoma;
MESO	Mesothelioma;
LIHC	Liver hepatocellular carcinoma;
LUAD	Lung adenocarcinoma;
LUSC	Lung squamous cell carcinoma;
PAAD	Pancreatic adenocarcinoma;
PRAD	Prostate adenocarcinoma;
PCPG	Pheochromocytoma and Paraganglioma;
READ	Rectum adenocarcinoma;
SARC	Sarcoma;
SKCM	Skin Cutaneous Melanoma;
LAML	Acute myeloid leukemia;
TGCT	Testicular Germ Cell Tumors;
THCA	Thyroid carcinoma;
THYM	Thymoma;
STAD	Stomach adenocarcinoma;
UCEC	Uterine Corpus Endometrial Carcinoma;
UCS	Uterine Carcinosarcoma;
UVM	Uveal Melanoma.

Supplementary Table 2. Clinical information of the 9 glioma patients.

Gender	Age	Tumor site	pathologic diagnosis
Female	37	right insular lobe	Low grade glioma(WHO grade II)
Man	64	left temporal lobe	Diffuse glial tumor(WHO grade III-IV)
Female	57	left parietal lobe	Glioblastoma(WHO grade IV)
Man	66	left parietal lobe	High grade glioma(WHO grade IV)
Man	48	left parietal lobe	Glioblastoma(WHO grade IV)
Man	58	left frontal lobe	WHO grade III-IV
Man	55	left frontal lobe	WHO grade III-IV
Man	52	left frontal lobe	Glioblastoma(WHO grade IV)
Man	63	left frontal lobe	Diffuse glial tumor (WHO grade III)

Supplementary Table 3. The top100 NID1-related genes in gliomas.

Gene Symbol	Gene ID	PCC
FMO1	ENSG00000010932.15	0.84
ABCA9	ENSG00000154258.16	0.81
EDARADD	ENSG00000186197.12	0.81
ENO1P1	ENSG00000244457.2	0.81
AC026904.1	ENSG00000233858.4	0.81
SCARA5	ENSG00000168079.16	0.80
C1QTNF9	ENSG00000240654.6	0.80
CILP	ENSG00000138615.5	0.80
KERA	ENSG00000139330.5	0.80
ADH1C	ENSG00000248144.5	0.80
SULT1E1	ENSG00000109193.10	0.80
RP11-567J20.1	ENSG00000253702.1	0.80
TNNT3	ENSG00000130595.16	0.79
CYGB	ENSG00000161544.9	0.79
FZD1	ENSG00000157240.3	0.79
FABP4	ENSG00000170323.8	0.79
RP11-199H2.2	ENSG00000282265.1	0.79
CAPN6	ENSG00000077274.8	0.79
DPPA3P3	ENSG00000270415.1	0.79
CYP1B1-AS1	ENSG00000232973.11	0.79
RP11-1K3.1	ENSG00000257281.1	0.79
PRG4	ENSG00000116690.11	0.79
CDC42EP5	ENSG00000167617.2	0.79
AP004372.1	ENSG00000238117.1	0.79
RP11-1260E13.1	ENSG00000262920.5	0.79
RN7SKP282	ENSG00000252633.1	0.79
UGT2B24P	ENSG00000249956.4	0.79
RP11-351A20.1	ENSG00000261818.1	0.79
AADACL2-AS1	ENSG00000242908.6	0.79
SNORA40	ENSG00000212579.1	0.79
RNU6-748P	ENSG00000207378.1	0.79
RP11-117D22.1	ENSG00000226938.1	0.79
P2RY10P2	ENSG00000232168.2	0.79
RNA5SP307	ENSG00000199733.1	0.79
RP11-204N11.2	ENSG00000258379.1	0.79
RP11-336A10.7	ENSG00000228685.1	0.79
SRP68P1	ENSG00000266129.1	0.79
AC011523.2	ENSG00000267968.1	0.79
RP11-146N18.1	ENSG00000267134.1	0.79
ZNF736P11Y	ENSG00000215537.3	0.79
RP11-259O2.2	ENSG00000248597.1	0.79
ZBTB8OSP1	ENSG00000183432.6	0.79
AC011193.1	ENSG00000225582.1	0.79
CTLA4	ENSG00000163599.14	0.79
CERS3	ENSG00000154227.13	0.78
RP11-401O9.4	ENSG00000273388.1	0.78
PRAC1	ENSG00000159182.4	0.78
RP11-359G22.2	ENSG00000230400.1	0.78

DCSTAMP	ENSG00000164935.6	0.78
AC093627.11	ENSG00000239715.1	0.78
AC064834.1	ENSG00000224099.1	0.78
RP11-392O17.1	ENSG00000228536.1	0.78
RP11-89H19.1	ENSG00000205537.2	0.78
AP000998.2	ENSG00000227330.1	0.77
RP11-401O9.3	ENSG00000264067.1	0.77
RP11-863P13.3	ENSG00000261327.4	0.77
PA2G4P1	ENSG00000237828.1	0.77
